# RECRUITMENT FOR A COMMUNITY-BASED SENSOR TECHNOLOGY PROGRAM: CHALLENGES AND SUCCESSES

**DOI:** 10.1093/geroni/igad104.0313

**Published:** 2023-12-21

**Authors:** Amy Vogelsmeier, Ashley Roberts, Suzette Bacon, Elizabeth Curtis, Erin Robinson, Blaine Reeder, Lori Popejoy

**Affiliations:** University of Missouri, Columbia, Missouri, United States; Sinclair School of Nursing, University of Missouri, Columbia, Missouri, United States; Sinclair School of Nursing, University of Missouri, Columbia, Missouri, United States; Sinclair School of Nursing, University of Missouri, Columbia, Missouri, United States; University of Missouri School of Social Work, Columbia, Missouri, United States; Sinclair School of Nursing, University of Missouri, Columbia, Missouri, United States; University of Missouri, Columbia, Missouri, United States

## Abstract

Eligibility criteria for the ASSETs for AIP program included older and disabled adults who were one-year post-discharge from the Missouri’s Show-Me Home program and who lived within a designated service area. The service area was comprised primarily of rural Missouri counties within the central I-70 corridor with smaller urban centers retained while larger urban areas were excluded. The goal was to enroll 33 people in year one, with an overall goal to enroll 100 individuals over the three-year project period. A client list was provided to the ASSETs for AIP team based on contact information collected at client discharge from the Show-Me Home Program. Despite diligent efforts, the team encountered several recruitment challenges including: 1) obtaining current contact information for individuals 12 months post-program discharge, 2) working within a limited-service area, and 3) establishing client trust in a new, unknown program upon initial contact. Successful recruitment strategies included 1) partnering with state and local agencies to retrieve up-to-date client contact information, 2) reducing the time from program discharge to facilitate contact and align better with client needs, 3) extending the service area beyond mid-Missouri, and 4) sending out postcard-mailers to potential clients that provided a clear linkage between Show-Me Home program, ASSETs for AIP, and the University of Missouri. A connection to the University was particularly important because the University has a statewide reputation for providing services to our citizens. Within the past 6 months, our enrollment has increased by 225%, with successful recruitment ongoing.

